# Factors Associated with Amblyopia Among the Elderly in the CA Medicare Population

**DOI:** 10.3390/medicina62101956

**Published:** 2026-10-10

**Authors:** Ricardo Oregon Guzman, Victoria L. Tseng, Ken Kitayama, Fei Yu, Anne L. Coleman

**Affiliations:** 1 David Geffen School of Medicine, University of California, Los Angeles, Los Angeles, CA 90095, USA; roregonguzman@mednet.ucla.edu; 2 Department of Ophthalmology, Stein and Doheny Eye Institutes, University of California, Los Angeles, Los Angeles, CA 90095, USA; 3 Department of Biostatistics, Fielding School of Public Health, University of California, Los Angeles, Los Angeles, CA 90095, USA; 4 Department of Epidemiology, Fielding School of Public Health, University of California, Los Angeles, Los Angeles, CA 90095, USA

**Keywords:** amblyopia, medicare, Social Vulnerability Index, health disparities, elderly population

## Abstract

*Background and Objectives*: Amblyopia is traditionally considered a pediatric condition; however, it may persist into adulthood and remain underrecognized. We examined the associations between sociodemographic and systemic factors and claims-documented amblyopia prevalence in the 2019 California (CA) Medicare population. *Materials and Methods*: This cross-sectional study used 2019 CA Medicare claims data for 2,649,107 beneficiaries aged ≥65 years, with Part A and B coverage, and valid ZIP codes for Social Vulnerability Index (SVI) values. Amblyopia was identified using ICD-10-CM diagnosis codes (H53.0x) in Medicare claims. Exposures included age, sex, race and ethnicity, systemic disease burden measured by Charlson Comorbidity Index (CCI) score, and Centers for Disease Control and Prevention SVI quartile. Logistic regression assessed associations between these factors and amblyopia diagnosis; descriptive statistics characterized refractive error and strabismus distribution by amblyopia status. *Results*: Amblyopia was present in 8460 (0.3%) beneficiaries. The largest proportions were aged 65–69 (n = 737,527; 27.8%), identified as female (n = 1,509,401; 57.0%), and were non-Hispanic White (n = 1,660,167; 62.7%). In adjusted models, increased odds of amblyopia were associated with ages 70–84 years, with the highest odds at ages 75–79 (age 75–79 vs. 65–69 adjusted odds ratio [aOR]: 1.42, 95% CI: 1.33–1.51), female sex (aOR: 1.10, 95% CI: 1.05–1.15), and a higher CCI score (CCI score ≥ 5 vs. 0 aOR: 1.30, 95% CI: 1.21–1.40). Decreased odds were associated with age ≥ 85 years (aOR: 0.88, 95% CI: 0.81–0.95), racial and ethnic minoritization (Black vs. Non-Hispanic White aOR: 0.51, 95% CI: 0.44–0.59), and a higher SVI quartile (Q4 vs. Q1 aOR: 0.67, 95% CI: 0.62–0.71). *Conclusions*: Ages 70–84 years, female sex, and higher systemic disease burden were associated with increased likelihood of amblyopia, while age ≥ 85 years, racially and ethnically minoritized groups, and those with higher social vulnerability had a decreased likelihood of documented amblyopia. Further research is needed to examine factors affecting amblyopia screening, diagnosis, and treatment in conjunction with comorbid ocular diseases in the elderly, including whether these differences reflect disparities in healthcare access and diagnostic coding rather than true differences in prevalence.

## 1. Introduction

Amblyopia is a visual disorder that occurs in early childhood when the normal communication between the eyes and the visual cortex is disrupted, leading the brain to suppress the weaker eye and rely on the stronger one, hence being colloquially referred to as a “lazy eye” [1,2]. This can lead to permanent vision loss without early correction and is the leading cause of monocular vision loss in children, with an estimated prevalence of 1–6% [1,2,3]. Treatment for amblyopia is most effective when initiated early and is focused on addressing the underlying source of visual deprivation and correcting refractive error [1]. Early screening for refractive error and strabismus improves prevention and treatment outcomes [4]. Despite the importance of early intervention, disparities in access to treatment exist, with children from socioeconomically disadvantaged backgrounds being five times more likely to have residual amblyopia [5] and Black and Spanish-speaking individuals showing increased rates of persistent amblyopia [6]. While socioeconomic factors may help explain the differences in amblyopia outcomes, limited research has explored this association.

Amblyopia causes permanent vision loss in 2.9% of adults and, in some areas, accounts for 33% of all unilateral visual impairments in adult patients [1]. This condition not only impacts vision, but also leads to impairment of other aspects of an individual’s life. Prior studies have found that amblyopia may lead to reduced reading speeds, abnormal motor skills, reduced stereo acuity, as well as an impact on the ability to drive, play sports, and complete work tasks [2,3,5]. The cost of eye care and the compromise of career choices associated with having amblyopia as an adult leads to an estimated loss of USD 7.4 billion in gross domestic product [5,7]. As patients with untreated amblyopia age, the likelihood of developing retinal or other eye abnormalities in their healthy eye increases, which leads to an increased risk of bilateral visual impairment [4]. Adults with amblyopia suffer the consequences of not having early intervention in their condition and are then subject to decreased quality of life, high costs of eye care, and increased likelihood of developing eye abnormalities in their stronger eye. At present, there is limited understanding of the risk factors for vision loss due to amblyopia in adulthood. As such, the purpose of this study is to investigate the association between sociodemographic and systemic factors and amblyopia in the 2019 CA Medicare population.

## 2. Materials and Methods

**Figure 1:**
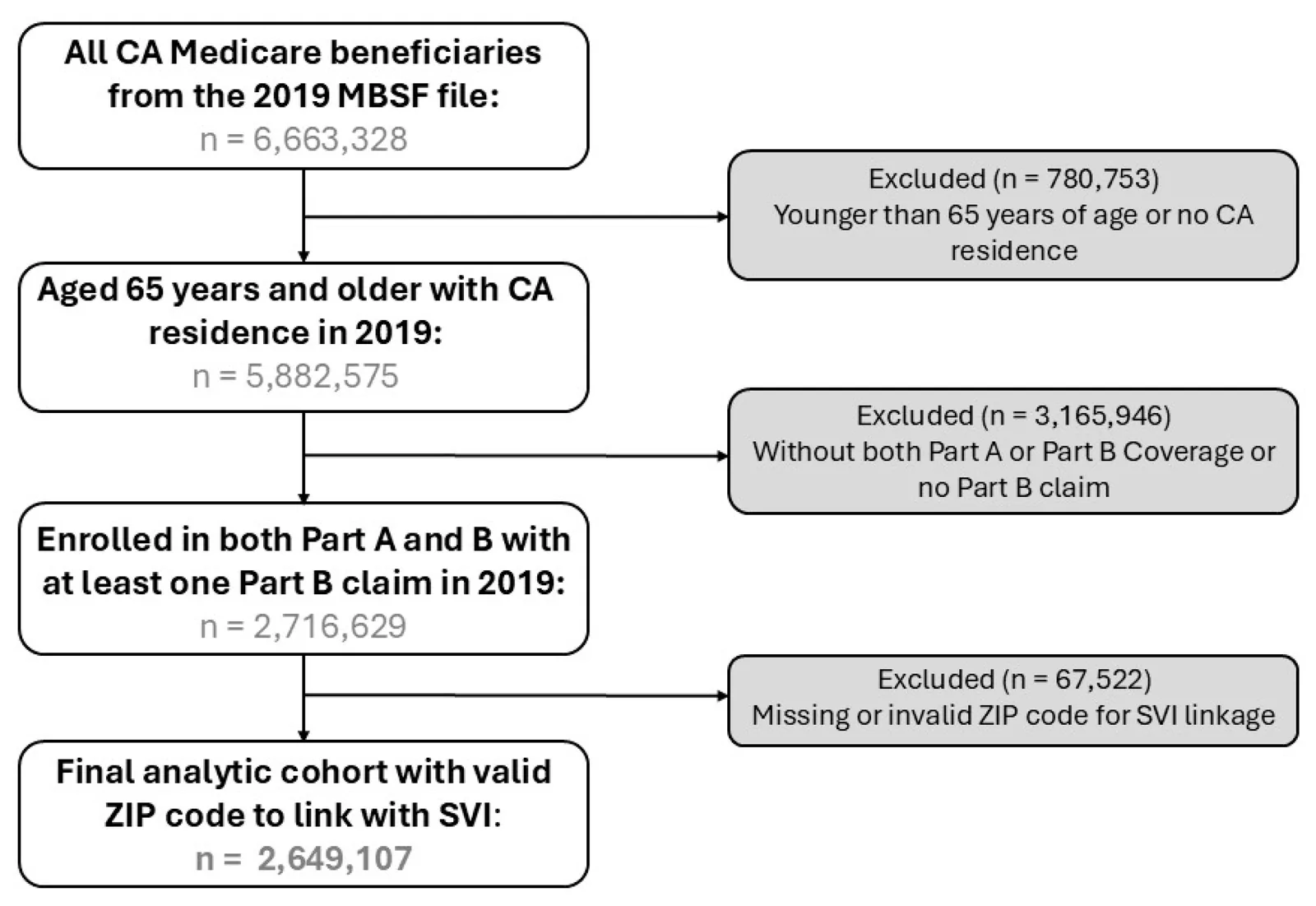
Flow diagram of study population selection.

This cross-sectional study included all fee-for-service 2019 CA Medicare beneficiaries who met the following criteria: age 65 or older, CA residence, enrollment in Medicare Parts A and B, with at least one Part B claim in 2019 (indicating active Part B coverage), and valid ZIP codes. Beneficiaries without Part A and B coverage in 2019, those without a Part B claim in 2019, and those with missing ZIP codes or ZIP codes that could not be linked to an SVI score were excluded. Selection of the study population is shown in Figure 1.

The outcome of interest was claims-documented amblyopia, defined as having one or more claims in 2019 with an International Classification of Diseases, Tenth Revision (ICD-10) diagnosis code for amblyopia (H53.0x, including H53.04-amblyopia suspect; see Appendix A for specific codes included). Suspected amblyopia was included because amblyopia is difficult to confirm in adults, in whom the history of childhood visual development is often unavailable. In older adults, amblyopia may instead be suspected on the basis of reduced best-corrected visual acuity without an identifiable ocular abnormality to explain it. Claims were identified in the Medicare dataset, a diagnosis code in any diagnosis position was counted, and no restriction was placed on provider specialty. Given this definition relies on diagnosis codes submitted for billing, the outcome reflects amblyopia documented in Medicare claims rather than clinically ascertained prevalence. Sociodemographic and systemic exposures of interest included age, sex, race and ethnicity, systemic disease burden measured by Charlson Comorbidity Index (CCI) score, and social vulnerability measured by Centers for Disease Control and Prevention Social Vulnerability Index (SVI) quartile.

Age was classified based on beneficiary age in 2019 and divided into 5-year groups: 65–69, 70–74, 75–79, 80–84, and ≥85 years. Sex was classified as male or female based on the Common Medicare Environment [8]. Race and ethnicity were classified as non-Hispanic White, Black, Hispanic/Latino, Asian, and Other based on the Research Triangle Institute (RTI) Race Code [9]. The CCI score is a measurement of systemic disease burden based on the presence or absence of 17 different comorbidities and reflects mortality risk within a patient population [10]. An increased CCI score reflects increased mortality risk and is scored 0, 1–2, 3–4, ≥5. Social vulnerability was defined based on the SVI score from data in the American Community Survey which is a subset of the US census. This was merged with the Medicare database based on beneficiary zip code of residence and classified in quartiles [11,12].

Comparison of baseline characteristics of beneficiaries with versus without amblyopia was carried out using chi-squared tests. *p*-values less than 0.05 were considered statistically significant. Univariable and multivariable logistic regression models were performed to assess associations between sociodemographic and systemic factors with the prevalence of amblyopia. A sensitivity analysis was performed for the main multivariable model using a logistic regression model with generalized estimating equations (GEE) to account for potential area level effects. The multivariable model included all study exposures in the same model. Descriptive statistics were performed for associated ocular diagnoses (myopia, hyperopia, astigmatism, any refractive error, and strabismus) in beneficiaries with and without amblyopia. Each refractive error type and strabismus was defined by the ICD-10 diagnosis code in the Part B claims (see Appendix A). All statistical analyses were performed using SAS version 9.4 (SAS Institute. Cary, NC, USA). This study was approved by the Institutional Review Board of the University of California, Los Angeles (IRB# 17-000914) and conformed to the requirements of the US Health Insurance Portability and Accountability Act of 1996.

## 3. Results

Table 1 outlines the baseline characteristics of the study population. The largest proportion of beneficiaries were aged 65–69 years (737,527/2,649,107; 27.8%), female (1,509,401/2,649,107; 57.0%), of non-Hispanic White race and ethnicity (1,660,167/2,649,107; 62.7%), and had a CCI score of 1–2 (922,072/2,649,107; 34.8%). There were 8,460/2,649,107 (0.3%) beneficiaries with a claims-documented diagnosis of amblyopia (Figure 1). There were statistically significant differences in all baseline characteristics in beneficiaries with versus without amblyopia (*p* < 0.05) (Table 1).

Table 2 describes the results from logistic regression analyses examining the odds of amblyopia by sociodemographic and systemic factors. In these multivariable models, the association between age and amblyopia was non-linear: compared with beneficiaries aged 65–69 years, the odds of amblyopia were higher among those aged 70–74 (adjusted odds ratio [aOR] = 1.29, 95% confidence interval [CI] = 1.21–1.37, *p* < 0.0001), 75–79 (aOR = 1.42, 95% CI = 1.33–1.51, *p* < 0.0001), and 80–84 years (aOR = 1.26, 95% CI = 1.18–1.36, *p* < 0.0001), with the highest odds at 75–79 years, and lower amond those aged ≥ 85 years (aOR = 0.88, 95% CI = 0.81–0.95, *p* = 0.0008). There was also increased odds of amblyopia in beneficiaries who were female (aOR = 1.1, 95% CI = 1.05–1.15, *p* < 0.0001) and had higher CCI scores (CCI score 5+ aOR = 1.3, 95% CI = 1.21–1.4, *p* < 0.0001). There was decreased odds of amblyopia in all racially and ethnically minoritized groups (Black aOR = 0.51, 95% CI = 0.44–0.59, *p* < 0.0001; Asian aOR = 0.55, 95% CI = 0.51–0.59, *p* < 0.0001; Hispanic/Latino aOR = 0.55, 95% CI = 0.50–0.59, *p* < 0.0001; Other aOR = 0.83, 95% CI = 0.75–0.93, *p* < 0.0001), and in increasing SVI Quartiles (Q4 aOR = 0.67, 95% CI = 0.62–0.71, *p* < 0.0001). When GEE modeling methods were applied, the overall magnitude and direction of findings did not change (Supplemental Table S1).

**Figure 2:**
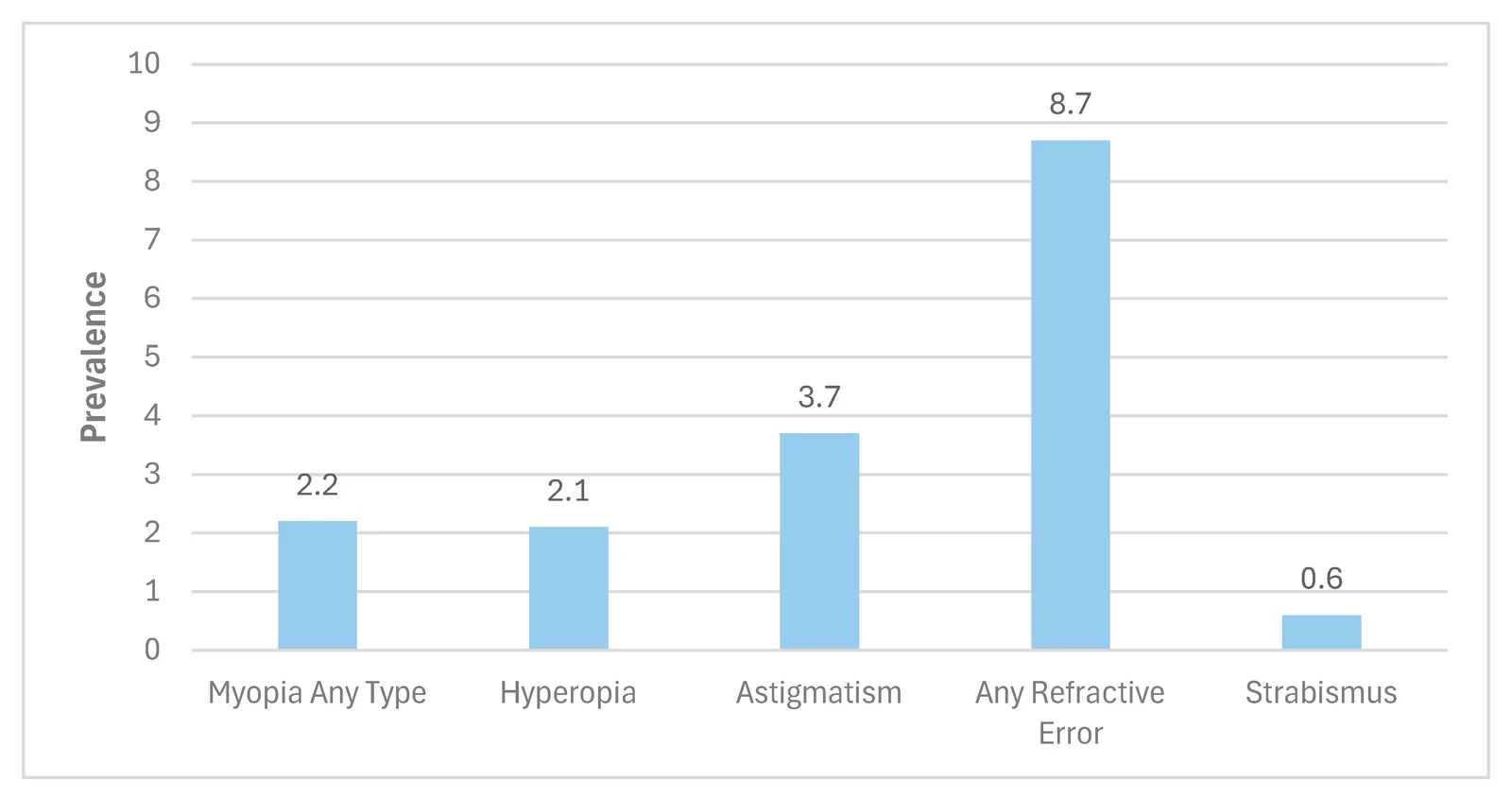
Prevalence of refractive errors and strabismus in total study population. Bar graph showing the prevalence of refractive errors, including myopia, hyperopia, astigmatism, and any refractive error, among the total study population of 2,649,107 California Medicare beneficiaries aged ≥65.

**Figure 3:**
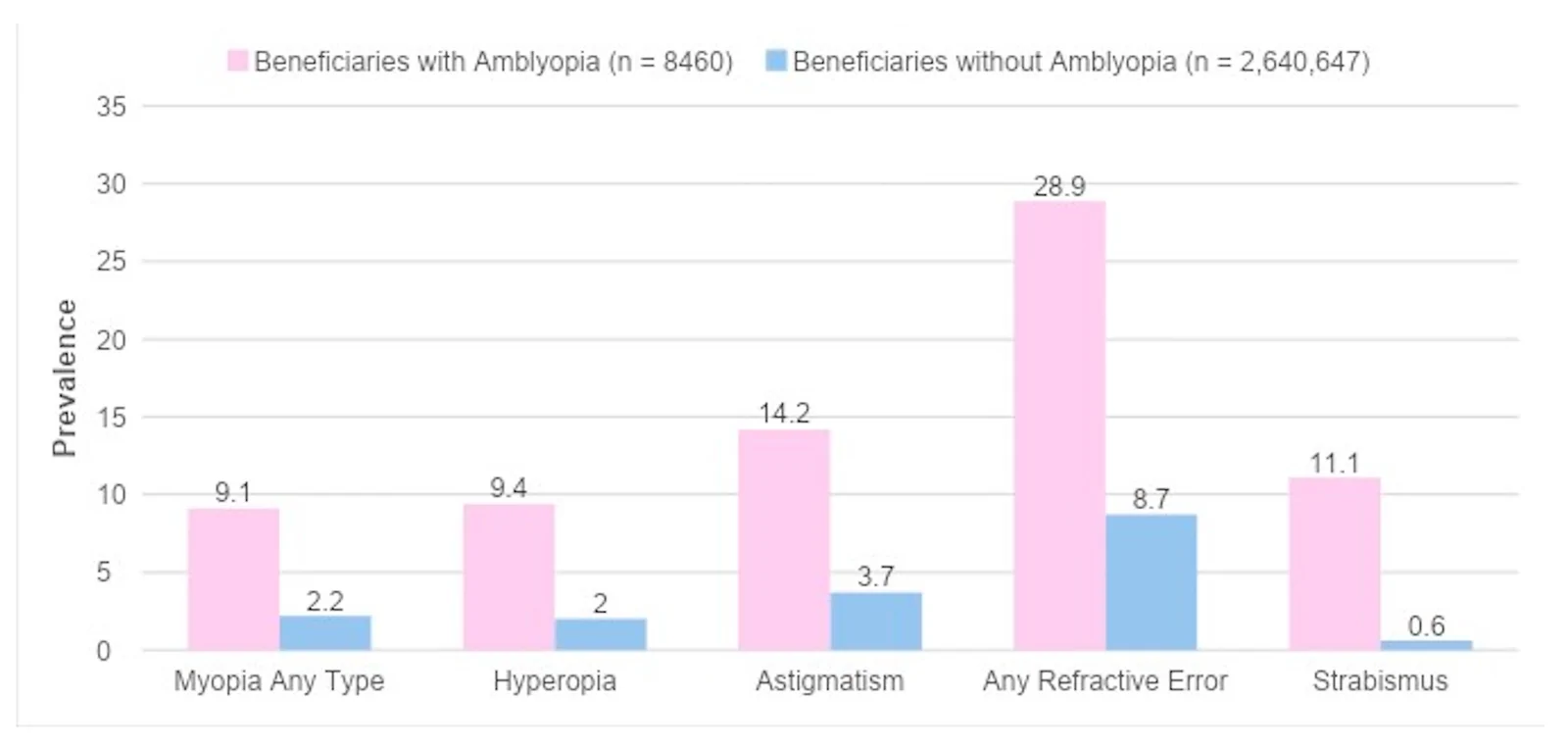
Prevalence of refractive errors and strabismus in beneficiaries with vs. without amblyopia. Bar graph comparing the prevalence of refractive errors, including myopia, hyperopia, astigmatism, and any refractive error, among California Medicare beneficiaries with and without amblyopia.

Figure 2 highlights the prevalence of refractive errors and strabismus in the total CA Medicare beneficiary population. Figure 3 compares the prevalence of refractive errors and strabismus in beneficiaries with versus without amblyopia. In beneficiaries with amblyopia, there was an increased prevalence of all refractive errors and strabismus compared to beneficiaries without amblyopia. The prevalence of refractive errors in beneficiaries with amblyopia was at least three times higher than in beneficiaries without amblyopia, and the prevalence of strabismus was approximately eighteen times higher in beneficiaries with amblyopia versus those without amblyopia. Among beneficiaries with amblyopia, astigmatism was the most commonly documented refractive error (14.2% vs. 3.7% in beneficiaries without amblyopia), followed by hyperopia (9.4% vs. 3.7% in beneficiaries without amblyopia), followed by hyperopia (9.4% vs. 2.0%) and myopia (9.1% vs. 2.2%) (Figure 3).

## 4. Discussion

In the 2019 CA Medicare population, ages 70–84 years (with the highest odds at 75–79 years), female sex, and greater systemic disease burden were associated with increased odds of a documented amblyopia diagnosis. In contrast, ages ≥85 years, racially and ethnically minoritized beneficiaries, and those with higher levels of social vulnerability had lower odds of amblyopia. Furthermore, beneficiaries with amblyopia had a higher prevalence of documented refractive error and strabismus diagnoses compared to beneficiaries without amblyopia.

Existing studies have established that amblyopia is the leading cause of monocular vision loss in both children and adults [13]. Studies by McConaghy, Miller, Fu, and Birch have found that amblyopia affects 1–6% of children and 1–2.9% of adults [1,2,14]. Furthermore, Attebo and colleagues found that amblyopia is a frequent cause of lifelong unilateral visual impairment in older populations [15]. A meta-analysis by Fu et al. estimated the global prevalence of amblyopia at 1.4%, with higher estimates in Europe (2.9%) and North America (2.4%) compared to Asia (1.1%) and Africa (0.7%) [14]. The prevalence of amblyopia in the present study was much lower than the global prevalence (0.3% vs. 1.4%). Several factors may explain this finding, including better screening and treatment in California, greater access to corrective lenses and specialty eye care in childhood, greater availability of early childhood health services, differences in how amblyopia is diagnosed across regions, and differences in how data is captured and recorded. Furthermore, prevalence estimates in the presents study are also likely underestimated given that providers may not commonly code for amblyopia during routine visits with Medicare patients. Prior studies have demonstrated that untreated amblyopia persisting into adulthood leads to visual impairment [4,15], emphasizing the need for improved diagnosis and treatment. This is particularly relevant given the findings of the current study, which found higher odds of documented amblyopia among beneficiaries aged 70–84 years compared with those aged 65–69 years, underscoring the importance of addressing amblyopia in adults. The association with age was not linear, as the odds of documented amblyopia were highest at 75–79 years and were lower among beneficiaries aged ≥ 85 years. Although this pattern could not be directly explained in the present study, possible explanations include greater eye care utilization in the eighth decade of life for age-related conditions, which may increase opportunities for amblyopia to be recorded, followed by reduced eye care utilization, competing medical priorities, or survival differences among the oldest beneficiaries. In addition, because amblyopia is partly a diagnosis of exclusion, reduced vision from common age-related ocular conditions may be misattributed to amblyopia in older adults, or amblyopia may go unrecorded when other ocular diagnoses are the focus of care.

The present study found that female sex was associated with increased odds of amblyopia in elderly adults. Prior studies in pediatric populations have not reported associations between sex and likelihood of amblyopia [14,16]. Our present findings of higher likelihood of amblyopia in female beneficiaries may be explained in part by our study of amblyopia in an elderly rather pediatric population. As women have a longer life expectancy [17], it is possible they are more likely to reach the older ages for which they will present for management of other age-related eye diseases and a diagnosis of amblyopia will be captured. Additionally, prior studies suggest that women have higher rates of healthcare utilization and a lower threshold for seeking medical care compared to men [18]. As a result, it is possible that women may have more opportunities for amblyopia to be diagnosed and coded, potentially contributing to the observed association in our study.

At present, there is limited data on the association between systemic disease burden (e.g., as estimated by the CCI score) and amblyopia. However, multiple studies exist that investigate the association between different components that make up the CCI score and ocular disease manifestations. These studies demonstrate that a wide range of systemic diseases are associated with increased incidence of ocular disease and ophthalmic manifestations [19,20]. Similarly, the association between higher CCI score and documented amblyopia in the present study may reflect more frequent healthcare use among beneficiaries with greater comorbidity, providing more opportunities for amblyopia to be diagnosed and coded. Since CCI score was measured in 2019, it reflects comorbidity in older adulthood rather than health status during the developmental period in which amblyopia arises, and this association should not be interpreted as causal or etiologic. It is possible that some beneficiaries with greater comorbidity also had systemic conditions in childhood that interfered with normal visual development or access to eye care [5]; however, childhood health status was not available in the present study, and this possibility remains speculative. These findings suggest that older adults with greater systemic disease burden may benefit from attention to ocular conditions, including amblyopia, as part of their care.

The association between race and ethnicity and amblyopia has been explored in several studies which have found that the prevalence of amblyopia varies across different racial and ethnic groups in the pediatric population. A multi-country, cross-sectional study by Xiao reported that the prevalence of amblyopia was highest among Hispanic children and lowest among children of African descent [16]. Similarly, the American Academy of Ophthalmology guidelines also highlight that Latinx children are more likely to develop amblyopia compared to Asian, White, or Black children [21,22]. Additionally, the Multi-Ethnic Pediatric Eye Disease Study found that amblyopia was more prevalent in Hispanic/Latino children compared to African American children early in childhood; however, they found that amblyopia prevalence remained stable with age [23]. In contrast, the present study found that there was a decreased odds of amblyopia in elderly adult beneficiaries from racially and ethnically minoritized backgrounds in the 2019 CA Medicare population. One possible explanation for this discrepancy is underdiagnosis and/or under-coding of amblyopia among adult beneficiaries from racially and ethnically minoritized backgrounds, although this could not be directly evaluated in the present study. It is possible that children from racially and ethnically minoritized backgrounds who receive fewer vision services or experience poorer treatment outcomes may carry amblyopia into adulthood without it being formally diagnosed or documented. The lower rates of amblyopia coding observed in these adults may reflect a continuation of disparities across the lifespan rather than a true lower prevalence. Another potential explanation is competing health priorities: adults from minoritized backgrounds have higher rates of undiagnosed chronic conditions such as diabetes mellitus and hypertension [24], which may shift clinical focus toward more acute or systemic concerns, leaving amblyopia unrecognized or undocumented. Additionally, well-documented disparities in U.S. eye care—which include later presentation, reduced disease monitoring, and worse visual outcomes among minoritized patients [21,22]—may contribute to missed opportunities for diagnosis. System-level factors may also play a role, as missed and delayed diagnoses in primary care have been linked to incomplete history-taking, inadequate physical examination, failure to pursue appropriate diagnostic testing, and insufficient consideration of differential diagnoses [25]. Together, these potential mechanisms suggest that clinical demands and structural factors in healthcare delivery could contribute to lower documentation of amblyopia in racially and ethnically minoritized patients. Given that healthcare access, eye care utilization, and clinical examination findings were not measured in the present study, these explanations should be considered hypotheses that warrant further investigation, including studies with clinical ascertainment of amblyopia across racial and ethnic groups.

Limited data exists on the association between amblyopia and SVI; however, several studies have examined the impact of sociodemographic factors on the prevalence, diagnosis, and treatment outcomes of amblyopia. The American Academy of Ophthalmology emphasizes that barriers to care and socioeconomic factors contribute to disparities in amblyopia treatment outcomes, emphasizing the importance of timely screening, comprehensive eye examinations, and optical correction to mitigate these disparities [21,22]. A study by Matsunaga and associates found that younger age at diagnosis, higher household income, and higher Childhood Opportunity Index scores (a measure of the quality of resources and conditions in every neighborhood in the United States) were associated with better amblyopia treatment outcomes [6]. Additionally, a study by Zhang et al. using the All of Us Research Program found that adults with amblyopia were more likely to be unemployed, have Medicaid insurance, and report lower household incomes, suggesting long-term socioeconomic consequences [26]. In contrast, the present study found that beneficiaries in higher SVI quartiles had lower odds of amblyopia. This finding may reflect differences in healthcare access, eye care utilization, and diagnostic coding among beneficiaries living in more socially vulnerable areas rather than a true lower prevalence of amblyopia. However, these factors were not directly measured in the present study due to inability to accurately capture these concepts with claims. In addition, SVI was calculated based on the census ACS data in 2018 and does not capture social or economic conditions during childhood, when amblyopia develops. Prior studies have also identified higher rates of underdiagnosis in underserved populations, reinforcing the need for improved screening, diagnosis, and treatment strategies for individuals with greater social vulnerability [21].

Beneficiaries with documented amblyopia also had a higher prevalence of documented refractive error and strabismus than beneficiaries without amblyopia, though based on the claims-based nature of the present study, these conditions should be interpreted as associated ocular diagnoses rather than confirmed underlying causes of childhood amblyopia. Among beneficiaries with amblyopia, astigmatism was the most commonly documented refractive error, which is consistent with pediatric studies identifying significant astigmatism as an amblyogenic risk factor [22], although age-related changes in astigmatism, including those associated with cataract and cataract surgery, may also contribute in older adults. Strabismus showed the greatest relative difference, which may reflect longstanding strabismus associated with strabismic amblyopia. Notably, the prevalence of documented refractive error in the overall study population was lower than would be expected in older adults. This likely reflects under-recording of refractive error in Medicare claims, as routine refraction is generally not a covered Medicare benefit, and suggests that the prevalence of refractive error in both groups is underestimated.

The strength of this study lies in its use of the 2019 CA Medicare beneficiaries, which is a large and diverse population. However, several limitations must be acknowledged. First, there is potential for misclassification and underestimation of true prevalence of amblyopia given the reliance on ICD-10 diagnosis codes, as amblyopia may not always be documented in claims data. Beneficiaries with amblyopia that was present but not coded during 2019 would not have been identified by our case definition. Varying coding practices by physicians may also contribute to underdiagnosis in minoritized populations. Although the higher prevalence of refractive error and strabismus among beneficiaries with amblyopia is consistent with the expected clinical profile of amblyopia, refractive error is likely under-recorded in Medicare claims, and this finding does not exclude misclassification. Second, differences in healthcare use and access to eye care could not be fully accounted for. Although beneficiaries had at least one Part B claim, we did not adjust for the number of healthcare visits, because a count of claims treats clinically different encounters (e.g., emergency department visits and routine office visits) as equivalent, and because eye care visits may partly result from an amblyopia diagnosis, adjustment for them could bias the estimates. We also did not restrict analyses to beneficiaries with an eye care encounter, because eye care visits cannot be reliably identified in claims data; eye care providers may bill general evaluation and management codes that are not specific to eye care. As a result, we could not determine the extent to which the lower odds of documented amblyopia among racially and ethnically minoritized beneficiaries and those with higher SVI reflect differences in access to care and diagnostic coding rather than true differences in prevalence. Future studies using validated measures of eye care utilization or clinical examination data are needed to address this question. Third, this was a cross-sectional study which limits the ability to infer causality. Although we can identify associations between variables, we are not able to establish causation since the data is collected at the single point of the claim submission. In addition, CCI score and SVI were measured in 2019 and 2018, respectively, and reflect beneficiaries’ comorbidity burden and area of residence in older adulthood rather than exposures during early childhood, when amblyopia develops; therefore, the observed associations should not be interpreted as etiologic. Lastly, although the 2019 CA Medicare beneficiary population is large and diverse, the findings of this study are not generalizable to all populations as the study population is an elderly population and may not fully represent the broader U.S. population.

## 5. Conclusions

This study identified ages 70–84 years, female sex, and increased systemic disease burden as factors associated with increased odds of documented amblyopia among the 2019 CA Medicare population, while age ≥ 85 years, racial and ethnic minoritization, and higher social vulnerability were associated with decreased odds. Since amblyopia was identified from Medicare claims, these findings may reflect differences in healthcare access and diagnostic coding rather than true differences in prevalence. This study also found a higher prevalence of documented refractive error and strabismus in individuals with amblyopia. These findings highlight the need for further research to elucidate barriers to screening, diagnosis, and treatment, particularly in vulnerable and marginalized groups. Future studies should investigate how social determinants of health, healthcare access, and systemic comorbidities interact to influence amblyopia prevalence and outcomes, thereby informing public health strategies aimed at reducing vision-related disparities and improving care across the lifespan.

## Figures and Tables

**Table 1:** Baseline demographic characteristics of California Medicare beneficiaries (2019).

	Total Population (n = 2,649,107)		Beneficiaries with Amblyopia (n = 8460)		Beneficiaries Without Amblyopia (n = 2,640,647)		
	n	%	n	%	n	%	*p*-Value
Age in years							
65–69	737,527	27.8	1903	22.5	735,624	27.9	<0.0001
70–74	663,219	25	2334	27.6	660,885	25	
75–79	486,942	18.4	1938	22.9	485,004	18.4	
80–84	346,688	13.1	1234	14.6	345,454	13.1	
≥85	414,731	15.7	1051	12.4	413,680	15.7	
Sex							
Male	1,139,706	43	3540	41.8	1,136,166	43	0.0283
Female	1,509,401	57	4920	58.2	1,504,481	57	
Race/Ethnicity							
Non-Hispanic White	1,660,167	62.7	6432	76	1,653,735	62.6	<0.0001
Black	113,255	4.3	201	2.4	113,054	4.3	
Asian	343,166	13	694	8.2	342,472	13	
Hispanic/Latino	419,627	15.8	775	9.2	418,852	15.9	
Other	112,892	4.3	358	4.2	112,534	4.3	
CCI Score ^a^							
0	909,600	34.3	2599	30.7	907,001	34.4	<0.0001
1–2	922,072	34.8	3013	35.6	919,059	34.8	
3–4	456,230	17.2	1629	19.3	454,601	17.2	
5+	361,205	13.6	1219	14.4	359,986	13.6	
SVI ^b^ Quartile							
Q1	647,563	24.4	2610	30.9	644,953	24.4	<0.0001
Q2	683,427	25.8	2415	28.6	681,012	25.8	
Q3	669,276	25.3	2028	24	667,248	25.3	
Q4	648,841	24.5	1407	16.6	647,434	24.5	

^a^ Charlson Comorbidity Index. ^b^ Social Vulnerability Index.

**Table 2:** Crude and adjusted logistic regression models for odds of amblyopia by sociodemographic and systemic factors.

	Crude Model			Adjusted Model ^a^		
Age Group	Odds Ratio	95% CI	*p*-Value	Odds Ratio	95% CI	*p*-Value
65–69	Reference					
70–74	1.36	1.28–1.45	<0.0001	1.29	1.21–1.37	<0.0001
75–79	1.54	1.45–1.65	<0.0001	1.42	1.33–1.51	<0.0001
80–84	1.38	1.28–1.48	<0.0001	1.26	1.18–1.36	<0.0001
85+	0.98	0.91–1.06	0.6402	0.88	0.81–0.95	0.0008
Sex						
Male	Reference					
Female	1.05	1.01–1.10	0.0284	1.1	1.05–1.15	<0.0001
Race/Ethnic Group						
Non-Hispanic White	Reference					
Black	0.46	0.40–0.53	<0.0001	0.51	0.44–0.59	<0.0001
Asian	0.52	0.48–0.56	<0.0001	0.55	0.51–0.59	<0.0001
Hispanic/Latino	0.48	0.44–0.51	<0.0001	0.55	0.5–0.59	<0.0001
Other	0.82	0.74–0.91	0.0002	0.83	0.75–0.93	0.0009
CCI ^b^ Score						
0	Reference					
1–2	1.14	1.09–1.21	<0.0001	1.18	1.12–1.25	<0.0001
3–4	1.25	1.18–1.33	<0.0001	1.31	1.23–1.4	<0.0001
5+	1.18	1.10–1.27	<0.0001	1.3	1.21–1.4	<0.0001
SVI ^c^ Quartile						
Q1	Reference					
Q2	0.88	0.83–0.93	<0.0001	0.91	0.86–0.96	0.0011
Q3	0.75	0.71–0.80	<0.0001	0.82	0.78–0.87	<0.0001
Q4	0.54	0.50–0.57	<0.0001	0.67	0.62–0.71	<0.0001

^a^ Adjusted model controls for study covariates. ^b^ Charlson Comorbidity Index. ^c^ Social Vulnerability Index.

## Data Availability

The data that support the findings in this study are available from the Centers for Medicare & Medicaid Services (CMS) but restrictions apply to the availability of these data, which were used under a data use agreement for the current study, and not publicly available. Data are available from the authors upon reasonable request and with permission of CMS.

## Supplementary Materials

The following supporting information can be downloaded at: https://www.mdpi.com/article/10.3390/medicina62101956/s1, Table S1: Generalized Estimating Equation Analysis of Factors Associated With Amblyopia.

## Appendix A

Amblyopia (ICD10 Codes: H53.00—unspecified amblyopia, H53.01—deprivation amblyopia, H53.02—refractive amblyopia, H53.03—strabismic amblyopia, H53.04—amblyopia suspect)

Refractive Errors

- Myopia (ICD10 Code: H52.1x)
- Hyperopia (ICD10 Code: H52.0x)
- Astigmatism (ICD10 Code: H52.2x)
- Any Refractive Error (ICD10 Code: H52.x)
- Any Strabismus (ICD10 Code: H50.x)
