# Supplementary material for: Factors Associated with Amblyopia Among the Elderly in the CA Medicare Population
Source: Medicina (Kaunas). 2026 Oct 10;62(10):1956. doi: 10.3390/medicina62101956 (PMC13706916; doi:10.3390/medicina62101956)
Supplement: Supplementary file 1 [file medicina-62-01956-s001.zip › medicina-4591050-supplementary.pdf]

Supplemental Table S1. Generalized Estimating Equation Analysis of Factors Associated With Amblyopia.

		Odds Ratio	95% Confidence Limits	P-value
Age				
	65 – 69 years old	Ref	Ref	Ref
	70 – 74 years old	1.28	1.20 - 1.36	<0.001
	75 – 79 years old	1.41	1.32 - 1.51	<0.001
	80 – 84 years old	1.25	1.15 - 1.35	<0.001
	85+ years old	0.86	0.79 - 0.93	<0.001
Sex				
	Male	Ref	Ref	Ref
	Female	1.09	1.05 - 1.14	<0.001
Race				
	White	Ref	Ref	Ref
	Black	0.53	0.46 - 0.60	<0.001
	Asian	0.55	0.51 - 0.60	<0.001
	Hispanic	0.56	0.52 - 0.61	<0.001
	Other	0.82	0.72 - 0.93	0.002
CCI				
	0	Ref	Ref	Ref
	1 – 2	1.18	1.12 - 1.25	<0.001
	3 – 4	1.32	1.23 - 1.41	<0.001
	5+	1.31	1.22 - 1.41	<0.001
SVI				
	Q1	Ref	Ref	Ref
	Q2	0.91	0.85 - 0.98	0.02
	Q3	0.82	0.75 - 0.88	<0.001
	Q4	0.65	0.59 - 0.72	<0.001
